# Cholinergic Transactivation of the EGFR in HaCaT Keratinocytes Stimulates a Flotillin-1 Dependent MAPK-Mediated Transcriptional Response

**DOI:** 10.3390/ijms16036447

**Published:** 2015-03-20

**Authors:** Sina Kühne, Wymke Ockenga, Antje Banning, Ritva Tikkanen

**Affiliations:** Institute of Biochemistry, Medical Faculty, University of Giessen, Friedrichstrasse 24, D-35392 Giessen, Germany; E-Mails: Sina.Kuehne@biochemie.med.uni-giessen.de (S.K.); Wymke.Ockenga@biochemie.med.uni-giessen.de (W.O.); Antje.Banning@biochemie.med.uni-giessen.de (A.B.)

**Keywords:** acetylcholine, muscarinic receptor, mitogen-activated protein kinase, cell proliferation, receptor tyrosine kinases, growth factor, keratinocytes, gene regulation

## Abstract

Acetylcholine and its receptors regulate numerous cellular processes in keratinocytes and other non-neuronal cells. Muscarinic acetylcholine receptors are capable of transactivating the epidermal growth factor receptor (EGFR) and, downstream thereof, the mitogen-activated protein kinase (MAPK) cascade, which in turn regulates transcription of genes involved in cell proliferation and migration. We here show that cholinergic stimulation of human HaCaT keratinocytes results in increased transcription of matrix metalloproteinase MMP-3 as well as several ligands of the epidermal growth factor family. Since both metalloproteinases and the said ligands are involved in the transactivation of the EGFR, this transcriptional upregulation may provide a positive feed-forward loop for EGFR/MAPK activation. We here also show that the cholinergic EGFR and MAPK activation and the upregulation of MMP-3 and EGF-like ligands are dependent on the expression of flotillin-1 which we have previously shown to be a regulator of MAPK signaling.

## 1. Introduction

Aside from its important role as a neurotransmitter, acetylcholine (ACh) also plays a major role in the communication and signal transduction in numerous non-neuronal cell types (reviewed in [1,2,3]). In many organs, various cells are capable of both synthesizing and secreting ACh, and they express both muscarinic and nicotinic subtypes of ACh receptors. The nicotinic ACh receptors (nAChRs) function as ion channels, whereas the muscarinic ACh receptors (mAChRs) are members of the large family of G protein coupled receptors (GPCRs). The five subtypes of mAChRs (designated as M_1_ to M_5_) transmit their signals mainly by means of heterotrimeric G proteins, which can be either inhibitory, resulting in reduced cyclic AMP amounts, or stimulatory, inducing a release of second messengers such as inositol 3,4,5-trisphosphate or diacylglycerol. Further downstream of these canonical signaling pathways, various protein kinases are activated that then regulate the function of numerous target molecules (for a review, see [4]).

In addition to their canonical signaling pathways, mAChRs are also capable of transactivating other receptors such as receptor tyrosine kinases. A well-characterized example is the epidermal growth factor receptor (EGFR) that is transactivated not only by stimulation of mAChRs, but also by other GPCRs [5,6,7,8,9,10], as well as by nicotinic AChRs [11]. The downstream signaling of the transactivated EGFR in turn is strongly coupled with the activation of the mitogen-activated protein kinase (MAPK) cascade that culminates in the activation of the MAP kinases of the extracellular signal regulated kinase (ERK) family, usually ERK1 and ERK2. As a response to EGFR/MAPK activation, ERK1/2 can phosphorylate and regulate either cytoplasmic substrate proteins or translocate into the nucleus and influence the activity of various transcription factors that actively regulate, e.g., cell proliferation (reviewed in [12]). Thus, mAChRs are principally capable of inducing a transcriptional response through MAP kinases by transactivation of the EGFR.

Transactivation of the EGFR by GPCRs is usually accomplished through a so-called “triple-membrane-passing-signal” pathway [13,14]. During this cascade, stimulation of a specific GPCR, e.g., a mAChR, is followed by the activation of a metalloproteinase that then proteolytically cleaves a membrane-bound precursor of a ligand of the EGF family. This results in extracellular release of an active ligand capable of activating the EGFR and thus inducing a downstream signaling response mediated by EGFR and the MAP kinase cascade. Depending on the cell type, the molecular details of this transactivation process show considerable flexibility (reviewed in [15,16,17]). In some cells, additional intracellular signaling cascades involving e.g., the Src kinases, phosphatidylinositol 3-kinase, or protein kinase C, are activated in parallel to the MAPK pathway, resulting in modulation of the downstream response (see, e.g., [8,18,19,20,21]). In addition, different metalloproteinases that usually belong either to the matrix metalloproteinase (MMP) or the ADAM (a disintegrin and metalloproteinase) family are activated. Furthermore, various ligands of the EGF-like family have been shown to be involved in EGFR transactivation, including the heparin-binding, EGF-like growth factor (HB-EGF), transforming growth factor α (TGFα), amphiregulin (AREG) and possibly epiregulin (EREG) [22,23,24,25].

In the human skin, ACh is an important regulator of keratinocyte proliferation and differentiation, and thus exerts an effect e.g., on wound healing (reviewed in [1]). Different layers of the epidermis express various mAChRs and exhibit different concentrations of ACh. In addition to the cholinergic regulation of keratinocyte physiology, EGFR and its ligands also play an important regulatory role in the epidermis [26]. We have earlier shown that in the human keratinocyte-like cell line HaCaT [27], cholinergic stimulation results in transactivation of the EGFR, which then induces a downstream response through the MAPK cascade [28]. In HaCaT cells, cholinergic MAPK activation is fully dependent on EGFR transactivation by mAChRs, mainly M_3_ and M_1_, but it does not involve Src kinases, protein kinase C or phosphatidylinositol 3-kinase. However, metalloproteinases that can be inhibited by the broad spectrum inhibitor, batimastat, are required for the EGFR ligand release [28].

Although EGFR transactivation and MAP kinases play such an important role in the regulation of cholinergic signaling in keratinocytes, surprisingly little is so far known about the transcriptional response that is induced by cholinergic MAPK activation. This study was carried out in order to shed more light on the regulation of MAPK-induced transcriptional response upon cholinergic stimulation of HaCaT keratinocytes. We especially studied the effect of MAPK activation on the transcription of specific proteins that are involved in EGFR transactivation, such as selected members of the MMP and ADAM families, as well as EGF-like ligands whose release has been shown to result in EGFR transactivation. Furthermore, since our earlier data have shown that the members of the flotillin protein family are important regulators of EGFR and MAPK signaling [29,30], we tested if flotillins also play a role in the cholinergic EGFR/MAPK activation.

We here show that certain metalloproteinases, especially MMP-3, and specific EGF-like ligands are transcriptionally upregulated in HaCaT cells upon cholinergic stimuli. This transcriptional activation depends on EGFR activity, whereas differences in the dependency on MAPK activity were detected in the case of EGF-like ligands. Furthermore, we show that the cholinergic MAPK activation and the downstream transcriptional response require the expression of flotillin-1. These data thus for the first time show that flotillin-1 can also affect the downstream signaling process of the mAChRs.

## 2. Results and Discussion

### 2.1. Results

Transactivation of the EGFR, which is induced by MMP/ADAM mediated proteolytic release of the ligands of the EGFR, is required for ERK activation upon cholinergic stimulus in HaCaT cells [28]. We here tested how cholinergic stimulation of HaCaT cells affects the expression of selected EGFR ligands and MMPs/ADAMs by measuring their transcript levels. We therefore especially focused on transcriptional effects that are mediated by the MAPK pathway. TGFα, HB-EGF, AREG and possibly EREG have been suggested to function in EGFR transactivation, and they are expressed in human keratinocytes [22,23,24,25]. Therefore, we measured the induction of the above-mentioned transcripts upon 2 h carbachol (CCh) stimulation with quantitative real-time PCR (qPCR) and analyzed the effect of inhibition of the mAChRs (by atropine), EGFR kinase activity (by PD 153035) and MEK1/2 kinases (by *U0126*) on their transcription*.*

**Figure 1:**
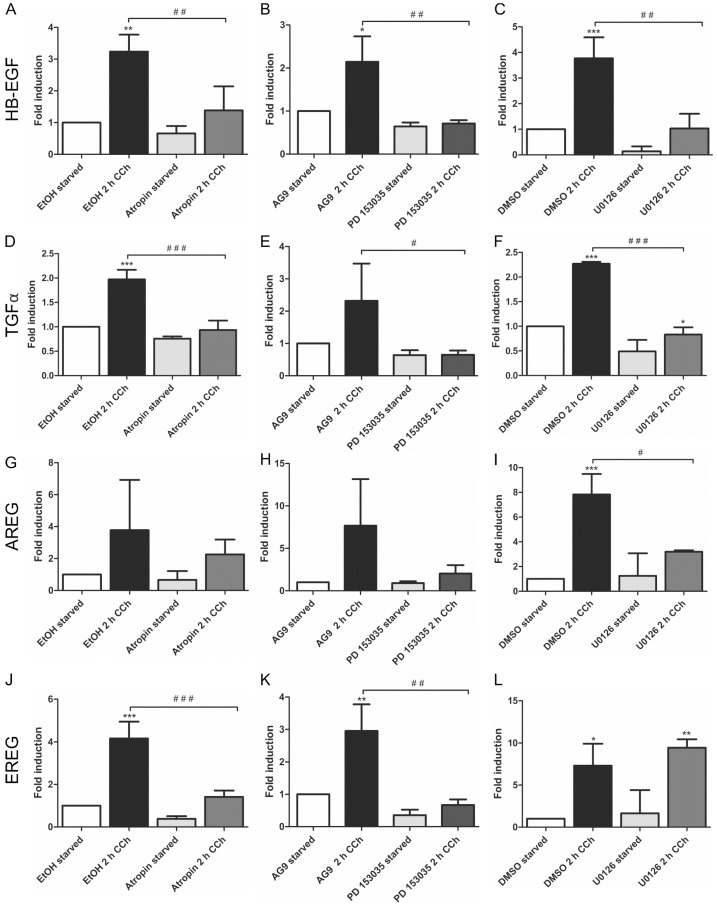
Transcriptional upregulation of specific ligands of the EGF family can be suppressed by inhibition of mAChRs, EGFR and MAP kinase signaling. Transcript levels of the EGF family ligands *HB-EGF* (**A**–**C**), *TGFα* (**D**–**F**), *AREG* (**G**–**I**) and *EREG* (**J**–**L**) were measured by qPCR in cells stimulated for 2 h with 100 µM CCh and treated with inhibitors of mAChRs (**A**,**D**,**G**,**J**: Atropin), EGFR (**B**,**E**,**H**,**K**: PD 153035) or MEK1/2 (**C**,**F**,**I**,**L**: U0126). Bars represent the mean ± SD of three independent experiments. Statistical analysis was performed with one-way or two-way ANOVA; *^,**#**^ *p* < 0.05, **^,**##**^ *p* < 0.01, ***^,**###**^ *p* < 0.001. * refers to comparison to unstimulated control, ^#^ to stimulated sample without inhibitor.

*HB*-*EGF* (Figure 1A–C) and *TGFα* (Figure 1D–F) were significantly upregulated upon CCh stimulation, and the induction of their transcripts by CCh was significantly reduced upon inhibition of mAChRs, EGFR and MEK. This shows that their cholinergic induction is dependent on mAChR-mediated transactivation of the EGFR and its downstream MAPK signaling. Similar data were obtained for *AREG*, but the induction of its transcript upon CCh varied to a large degree, preventing statistically significant differences in the data (Figure 1G–I). *EREG*, on the other hand, was significantly induced upon CCh, and its transcription was inhibited upon mAChR and EGFR but not MEK inhibition (Figure 1J–L). This suggests that in HaCaT cells, *EREG* may be induced by a mAChR and EGFR dependent pathway that does not involve MAP kinases. *EGF*, *betacellulin* and *epigen*, which are also involved in EGFR family signaling, were not upregulated by CCh stimulation (data not shown).

**Figure 2:**
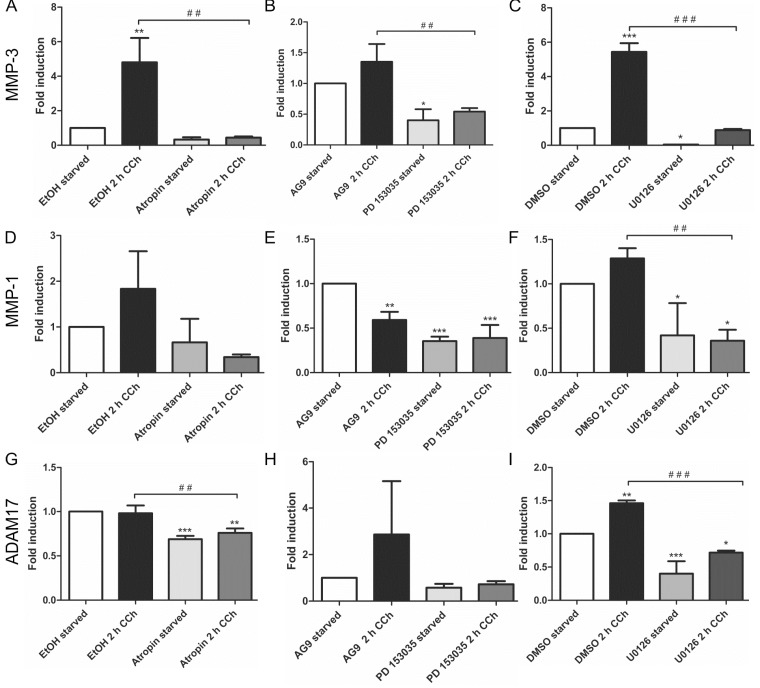
*MMP-3* is upregulated by cholinergic stimuli in a manner dependent on mAChRs, EGFR and MAP kinases. HaCaT cells were stimulated with 100 µM CCh for 2 h and treated or not with inhibitors of the mAChRs, EGFR or MEK1/2. Transcriptional regulation of *MMP-3* (**A**–**C**), MMP-1 (**D**–**F**) and *ADAM17* (**G**–**I**) was measured by qPCR. Bars represent the mean ± SD of three independent experiments. Statistical analysis was performed with one-way or two-way ANOVA; * *p* < 0.05, **^,**##**^ *p* < 0.01, ***^,**###**^ *p* < 0.001. * refers to comparison to unstimulated control, ^#^ to stimulated sample without inhibitor.

**Figure 3:**
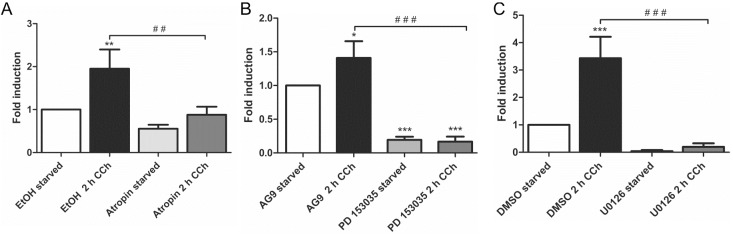
*Egr1* transcription is stimulated by CCh and repressed by inhibition of mAChRs (**A**), EGFR (**B**) and MAP kinases (**C**). Transcriptional induction of *Egr1* was measured by qPCR as stated in the legend of Figure 2. Bars represent the mean ± SD of three independent experiments. Statistical analysis was performed with one-way or two-way ANOVA; * *p* < 0.05, **^,**##**^ *p* < 0.01, ***^,**###**^ *p* < 0.001. * refers to comparison to unstimulated control, ^#^ to stimulated sample without inhibitor.

Our earlier findings have shown that a metalloproteinase that can be inhibited by the broad spectrum MMP/ADAM inhibitor, batimastat, mediates the release of EGF-like ligands during cholinergic EGFR transactivation [28]. We thus focused our analysis on MMPs/ADAMs that are inhibited by batimastat and/or have been implicated in the release of the EGF-like ligands studied above. Of the MMPs and ADAMs tested, only *MMP*-3 showed a systematic transcriptional induction upon CCh stimulation, which was significantly reduced upon mAChR, EGFR and MEK inhibition (Figure 2A–C). *MMP-1* was only modestly, but non-significantly induced by CCh, but its basal expression was blocked by all three inhibitors (Figure 2D–F), whereas *MMP-2*, *ADAM10* and *ADAM8* appeared not to be regulated by cholinergic stimuli (data not shown). The degree of induction of *ADAM17* varied largely between experiments, but its basal expression was reduced by atropine and the MEK inhibitor (Figure 2G,H). Finally, we tested the transcriptional induction of Early Growth Response Gene 1 (*Egr1*), whose expression is directly induced by MAPK-mediated signaling. Egr1 expression was significantly induced by CCh and significantly blocked upon inhibition of mAChRs, EGFR and MAPK signaling (Figure 3A–C), as expected from a MAPK regulated protein.

**Figure 4:**
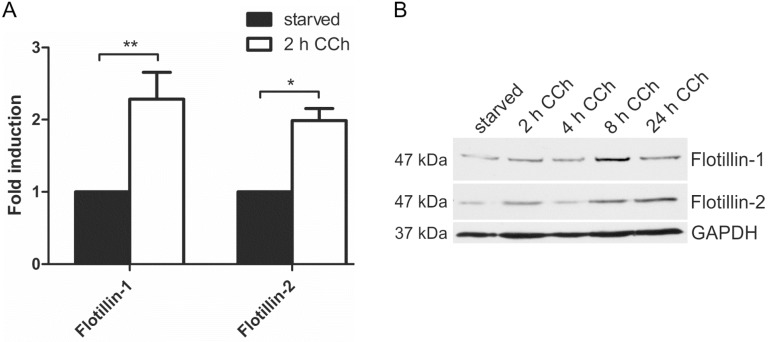
Cholinergic stimulation of HaCaT cells results in increased flotillin expression. HaCaT cells were stimulated with 100 µM CCh as indicated and the mRNA (**A**) and protein (**B**) levels of flotillins were determined. Bars in (**A**) represent the mean of 3 independent experiments. Statistical analysis was performed with one-way ANOVA; * *p* < 0.05, ** *p* < 0.01; (**B**) shows a representative Western blot for flotillins upon CCh stimulation.

Our previous data have shown that the members of the flotillin protein family, flotillin-1 and flotillin-2, are regulators of the MAPK signaling mode upon direct stimulation of the EGFR with EGF [29,31]. Furthermore, promoter activities of flotillin genes are under control of EGFR/MAPK signaling and the Egr1 transcription factor [32]. Since cholinergic ERK activation in HaCaT cells requires EGFR and results in Egr1 activation ([28] and Figure 3), we tested if flotillin expression would be induced by cholinergic stimulation of HaCaT cells. Indeed, 2 h CCh stimulation resulted in a significant increase in both flotillin-1 and flotillin-2 mRNA levels (Figure 4A). Accordingly, a moderate increase of flotillin-1 and flotillin-2 at the protein level was observed after 8 h CCh stimulation (Figure 4B). Thus, these data identify flotillins as novel genes that are regulated by cholinergic signaling in human HaCaT keratinocytes.

**Figure 5:**
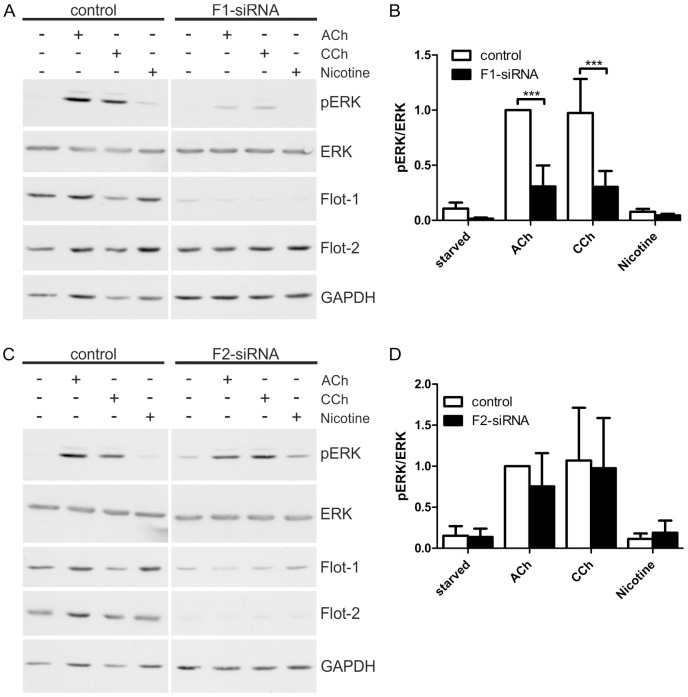
Flotillin-1 knockdown impairs cholinergic signaling towards ERK. HaCaT cells were depleted of flotillins by means of specific siRNAs. The cells were starved and stimulated with 100 µM ACh, 1 mM CCh or 100 µM nicotine for 30 min. (**A**,**C**): Equal amounts of protein were separated by SDS-PAGE, and the phosphorylation of ERK1/2 was analyzed; (**B**,**D**): The amount of pERK1/2 was determined by densitometric quantification and normalized to total ERK1/2. Data are shown relative to the ACh stimulated control cells. Bars represent the mean ± SD of five independent experiments. Statistical analysis was performed with two-way ANOVA; *** *p* < 0.001.

To further elucidate the role of flotillins in cholinergic ERK activation in HaCaT cells, we performed knockdown experiments for both flotillins using siRNA mediated depletion. A significantly reduced ERK activation was observed in flotillin-1 knockdown cells upon stimulation with ACh or CCh (Figure 5A,B), whereas flotillin-2 knockdown did not impair the cholinergic ERK activation (Figure 5C,D). It should be noted that flotillin-2 knockdown results in a moderate reduction of flotillin-1 protein levels (Figure 5C), which appears not to be sufficient to impair MAP kinase activation. Consistent with our previous data [28], nicotine did not induce any ERK activity even in the control cells, implicating that the nAChRs are not involved in MAP kinase activation in these cells.

**Figure 6:**
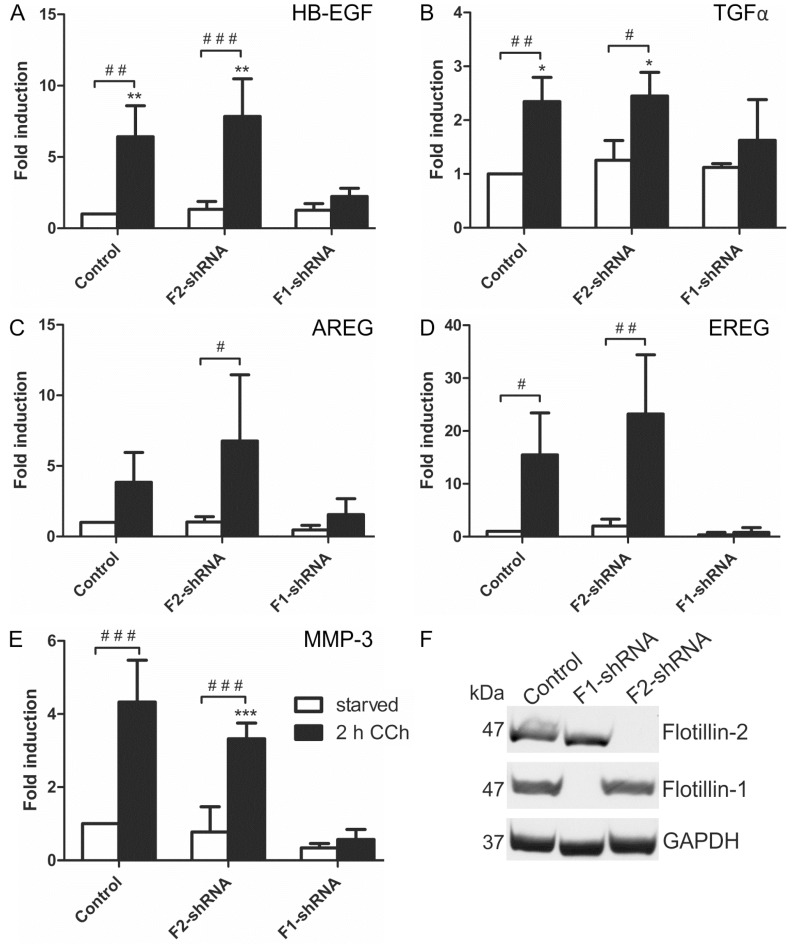
Flotillin-1 is required for CCh-induced transcriptional regulation of EGF family ligands and MMP-3. Stable flotillin-1 and flotillin-2 knockdown and control HaCaT cells were stimulated with 100 µM CCh for 2 h and the transcript levels of EGF family ligands (**A**–**D**) and MMP-3 (**E**) were determined; (**F**) Knockdown efficiency of flotillin-1 and flotillin-2 as detected by Western blot. Bars represent the mean ± SD of three independent experiments. Statistical analysis was performed with one-way or two-way ANOVA; *^,**#**^ *p* < 0.05, **^,**##**^ *p* < 0.01, ***^,**###**^ *p* < 0.001. * refers to comparison to unstimulated control cells, ^#^ to stimulated sample of the respective cell line.

According to the above data, flotillin-1 appeared to be important for the cholinergic activation of the MAPK pathway in HaCaT cells. Thus, we tested if the transcriptional induction of the EGFR ligands and MMP-3, which we above identified as target genes of cholinergic MAPK response, would also depend on flotillins. Indeed, transcriptional induction of mRNAs of all four EGFR ligands (*HB-EGF*, *TGFα*, *AREG* and *EREG*, Figure 6A–D) and of *MMP-3* (Figure 6E) was significantly impaired upon stable shRNA-mediated knockdown of flotillin-1 in HaCaT cells, whereas it was not affected by the knockdown of flotillin-2. Knockdown efficiencies are shown in Figure 6F. Thus, these findings identify flotillin-1 as a novel regulator of the downstream signaling and transcriptional response induced by cholinergic EGFR transactivation.

### 2.2. Discussion

Although the molecular mechanisms of transactivation of the EGFR by GPCRs are becoming increasingly well characterized, little is known about the transcriptional changes that take place downstream thereof. EGFR transactivated by the mAChRs in HaCaT cells efficiently signals downstream towards MAP kinases, which are known regulators of cell proliferation. The present study was carried out in order to characterize the transcriptional effects of cholinergic EGFR and MAPK activation on the expression of selected target genes. We rationalized that activation of the EGFR and MAP kinases after cholinergic stimulus may result in a transcriptional response that increases the expression of proteins participating in EGFR activation and cell proliferation. We thus especially focused on genes that are known to participate in GPCR-mediated EGFR transactivation, such as metalloproteinases of the MMP and ADAM families and EGF family ligands. Transcriptional induction of these genes upon a cholinergic stimulus was first analyzed in order to identify those genes that are upregulated upon CCh/mAChR activation. A further aim was to study if the cholinergic upregulation of these genes is dependent on EGFR and/or MAP kinases. We could show that HB-EGF, TGFα, AREG and EREG all are transcriptionally induced upon stimulation of the mAChRs in HaCaT cells, whereas betacellulin, epigen and EGF showed no upregulation upon CCh stimulation. Of the ADAMs/MMPs studied, only MMP-3 was clearly and reproducibly induced upon cholinergic stimulation of the cells. ADAM10, ADAM17, MMP-1 and MMP-2, however, were only occasionally upregulated, but their basal expression was frequently downregulated by inhibitors of the EGFR and MEK.

Very few studies have addressed the regulation of MMPs/ADAMs or EGFR ligands upon cholinergic stimuli. Xie *et al.* could show in H508 colon cancer cells that Ach-induced cell proliferation is mediated by EGFR/MAPK signaling [33]. Since M_3_ is the only mAChR expressed in these cells [7], it appears that this receptor subtype is involved in cholinergic EGFR transactivation in both H508 and HaCaT cells [28,33]. Xie *et al.* detected a transcriptional increase in MMP-1, MMP-7 and MMP-10, which was EGFR and MAPK dependent. However, in contrast to what we have shown here in HaCaT cells, MMP-3 was not upregulated or even expressed in H508 cells [33], suggesting that despite the involvement of the same mAChRs, the transcriptional responses are different. In contrast to cholinergic stimuli, upregulation of MMP expression by direct EGFR mediated signaling has been addressed by a number of studies that have mainly used direct stimulation with EGF. These studies have shown a cell type dependent upregulation of, e.g., MMP-1, -3, -7 and -11 [34,35,36] as well as of MMP-9 [37]. In addition, the expression of MMP-2, -9, -11, -13 and -17 is reduced in embryonic fibroblasts not expressing the EGFR [36], further suggesting a causality between EGFR signaling and MMP regulation.

Unfortunately, transcriptional regulation of EGF-like ligands by cholinergic stimuli has so far not been directly addressed. Xie *et al.* could detect the expression of TGFα, HB-EGF and AREG in H508 cells, whereas EGF, betacellulin, EREG and epigen were not expressed, and transcriptional regulation of the ligands was not studied [33]. In HaCaT cells, these seven ligands are expressed, although only four of them are transcriptionally regulated by cholinergic stimuli. Thus, it is possible that upregulation of a specific subset of EGF family ligands and metalloproteinases provides a positive feed-forward loop that strengthens cholinergic EGFR transactivation, as more of the activating ligand and possibly of the sheddase could become available. The existence of such a feed-forward loop was not directly shown in our study, but it should be addressed in future studies. EGF family ligands and MMPs are involved in important regulatory processes, e.g., matrix remodeling and differentiation (reviewed in [38,39,40]), and their upregulation may be part of a downstream mechanism that results, e.g., in changes in cell communication with its environment. As EGFR and MAPK signaling also regulate cell proliferation and migration in cancer cells, it will be important to characterize the mechanisms of cholinergic EGFR transactivation in detail, as crosstalk between GPCRs and EGFR has been shown to take place in numerous cancers [10,41,42].

Very little is known about the identity of the metalloproteinases and the shed ligands that are involved in cholinergic EGFR activation. It has previously been shown that in squamous cell carcinoma cells, CCh stimulation activates ADAM17, which then sheds AREG to activate EGFR signaling [43]. However, which ADAM/MMP is activated and which ligand will be released is dependent on the cell type, and most likely also on the mAChR involved [44]. At present, it is not clear which metalloproteinase mediates the cholinergic EGFR transactivation and which EGF-like ligand is released in HaCaT cells, which should be characterized in further studies.

We here could show that the cholinergic activation of the MAP kinase pathway in HaCaT cells is dependent on flotillin-1. This is consistent with our earlier data showing that EGF induced MAPK activation is severely impaired upon depletion of flotillin-1 [29]. Flotillin-2 depletion, however, did not result in reduced cholinergic ERK activation although flotillin-1 expression was also moderately reduced. Most likely, the remaining flotillin-1 was sufficient to support MAPK signaling under these experimental conditions. Taken together, these data identify flotillin-1 as the major regulator of EGFR/MAPK signaling among the two flotillin family members.

Our earlier findings have shown that flotillin-1 regulates EGF-induced EGFR/MAPK signaling at two levels [29]. On one hand, flotillin-1 is required for the clustering and phosphorylation of the EGFR at the plasma membrane. On the other hand, flotillin-1 also directly binds and scaffolds several proteins that participate in the MAPK cascade, including the MAP kinases ERK1/2 and their upstream activators, MEK1/2 [29]. The cholinergic MAPK activation in HaCaT cells proceeds through the mAChRs, metalloproteinase activation, EGF-like ligand release and finally EGFR activation [28]. Similarly to direct activation of EGFR by EGF [29], the cholinergic EGFR transactivation and its downstream signaling to MAP kinases also depends on flotillin-1. Consistently, the downstream transcriptional induction of most of the target genes associated with EGFR transactivation upon cholinergic stimulus was dependent on both EGFR and MAP kinase activity, as well as on flotillin-1 expression. Of the four EGF-like ligands that we found to be upregulated upon CCh stimulation, only EREG transcription was not inhibited upon MEK inhibition. This suggests that although enhanced EREG transcription requires EGFR activation, it might not be directly regulated by MAP kinases but by another signaling pathway induced by EGFR. However, flotillin-1 knockdown profoundly impaired the transcriptional induction of all four EGF-like ligands studied here, including EREG. This is very likely due to the fact that flotillin-1 knockdown also directly impairs EGFR activation [29], thus also preventing EREG induction upon cholinergic stimulus.

It is plausible that flotillins may also participate in cholinergic signaling steps preceding EGFR transactivation, such as activation of the mAChRs, as flotillins have previously been shown to be functionally associated with specific GPCRs. During melanoma metastasis, flotillin-2 regulates the expression of the GPCR “protease activated receptor 1” (PAR1), but it is not known if flotillins are also directly involved in PAR1 signaling [45]. In UTP activated cells, flotillins were shown to interact with the Gα_q_ subunit and to regulate signaling through the P2Y nucleotide receptor [46]. Since both M_1_ and M_3_ receptors can also signal through Gα_q_ subunits, it will be of interest to clarify the role of flotillins in canonical mAChR signaling. Considering the important role of EGFR/MAPK signaling and also of cholinergic signaling in cancer cells [47], our findings may also be relevant in dissecting the molecular mechanisms through which cholinergic stimuli can increase proliferation, and inhibit apoptosis, in malignant cells.

## 3. Experimental Section

### 3.1. Reagents and Antibodies

Cholinergic agonists and the mACh receptor inhibitor atropine were purchased from Sigma–Aldrich (Taufkirchen, Germany). The EGFR kinase inhibitor PD 153035 (Cat. 234490) and the non-inhibiting control compound AG9 (Cat. 658390) were obtained from Merck/Millipore (Darmstadt, Germany). The MEK1/2 inhibitor U0126 was from Cell Signaling (Frankfurt am Main, Germany). Rabbit polyclonal antibody against ERK2 and the mouse monoclonal antibody against phospho-ERK1/2 were purchased from Santa Cruz Biotechnology (Santa Cruz, CA, USA). For flotillin-1 and flotillin-2, monoclonal mouse antibodies from BD Transduction Laboratories (Franklin Lakes, NJ, USA) were used. A mouse monoclonal antibody against GAPDH was obtained from Abcam (Cambridge, UK). Secondary antibodies goat anti-mouse and goat anti-rabbit coupled to a horseradish peroxidase (HRP) were purchased from Dako (Glostrup, Denmark).

### 3.2. Cell Culture

Human immortalized keratinocyte (HaCaT) cells [27] were obtained from P. Boukamp (German Cancer Research Center, Heidelberg, Germany) and cultured in Dulbecco’s modified Eagle’s medium (DMEM, Gibco, Life Technologies, Carlsbad, CA, USA) with high glucose, 10% fetal calf serum, 100 U/mL penicillin and 100 μg/mL streptomycin. The medium was supplemented with 1% non-essential amino acids and 1 mM sodium pyruvate (all from Life Technologies). The cells were grown in a humidified incubator at 8% CO_2_ and 37 °C.

### 3.3. Flotillin Knockdown by siRNA and shRNA

For a transient knockdown of flotillins, two different siRNA duplexes (Stealth™ siRNA system; Life Technologies) were used. We have used the flotillin siRNAs in previous studies and shown that they do not produce any off-target effects and are specific for flotillins (see, e.g., [29,31,48]). As a control, siRNAs that do not recognize any human genes were used. The siRNAs were transfected in HaCaT cells with the Lipofectamine RNAiMAX Reagent (Life Technologies), and the cells were used for experiments 72 h post-transfection. For a stable knockdown of flotillins in HaCaT cells, we used the Mission Lentiviral shRNA system (Sigma–Aldrich), with shRNA sequences targeting human flotillin-1 or flotillin-2. Lentiviruses not targeting any human gene were used to establish a control cell line. We have earlier used these viruses to generate stable flotillin knockdowns in HeLa, MCF7 and MCF10A cells [48,49,50].

### 3.4. Cell Stimulation and Inhibitor Treatment

HaCaT cells were grown to 80%–100% confluency and serum-starved for 18–24 h before treatment with 1 mM CCh, 100 µM ACh or 100 μM nicotine for the indicated times. Control cells were treated with serum-free medium without any stimulant. For the inhibition of mAChRs, HaCaT cells were pretreated with 25 μM atropine for 30 min before CCh-stimulation. PD 153035 (EGFR kinase inhibitor, 1 μM) and AG9 (control, 1 μM) were preincubated for 5 min. The MEK1/2 inhibitor U0126 (10 μM) was added 2 h before stimulation.

### 3.5. Cell Lysis, Gel Electrophoresis and Western Blot

Cell pellets were lysed in lysis buffer (50 mM Tris–HCl pH 7.4, 150 mM NaCl, 2 mM EDTA, 1% Nonidet P-40) supplemented with protease inhibitor cocktail (Sigma–Aldrich) and 1 mM sodium orthovanadate/1 mM NaF. Protein concentration was measured with the BioRad Protein Assay reagent (BioRad, Munich, Germany). Equal protein amounts of the lysates were analyzed by SDS–polyacrylamide gel electrophoresis and Western blot. For antibody staining, the membranes were blocked with 5% non-fat dry milk in TBST (10 mM Tris, 150 mM NaCl, 0.05% Tween 20) and incubated over night at 4 °C with the indicated primary antibodies diluted in TBST. This was followed by incubation with HRP-conjugated secondary antibodies for 1 h at room temperature before development with Amersham™ ECL™ Western Blotting Detection Reagents (GE Healthcare, Buckinghamshire, UK).

### 3.6. RNA Isolation and RT-qPCR

Total RNA from HaCaT cells was isolated with peqGold TriFast reagent (Peqlab/VWR, Erlangen, Germany), after which a DNase I (10 U, NEB, Frankfurt am Main, Germany) digestion was performed. For qPCRs, 3 µg total RNA was reverse transcribed with 1.5 pM oligo(dT) primers and the M-MuLV reverse transcriptase (NEB). Real-time quantitative PCRs were performed using the CFX Connect Real-Time PCR Detection System (Bio-Rad, Hercules, CA, USA). The reactions were done as duplicates with 0.8 µL of 5-fold diluted cDNA in 9 μL iTaqTMUniversal SYBR Green Supermix (Bio-Rad). The qPCR primers were designed with PerlPrimer (Table 1). PCR products were quantified with the Δ*C*_t_-method. For normalization, the geometric mean of the reference genes Rpl13a, HPRT and Ywhaz was used.

### 3.7. Statistical Analysis

All experiments were performed at least three times. Data are expressed as mean ± S.D. Statistical comparisons between groups were made using one-way or two-way analysis of variance (ANOVA), as appropriate, using GraphPad Prism 5 (GraphPad Software, La Jolla, CA, USA). Values of *p* < 0.05 were considered significant (* or #), and values of *p* < 0.01 and *p* < 0.001 were defined very significant (** or ##) and highly significant (*** or ###), respectively.

**Table 1:** Primer sequences and annealing temperatures for the qPCR primers used in this study. All sequences are given in 5'–3' direction.

Gene	Primer Forward	Primer Reverse	*T*_Annealing_ (°C)
*cFos*	TGGTGAAGACCGTGTCAGGAG	TGATCTGTCTCCGCTTGGAGTG	60
*Dusp1*	GGAGGACAACCAGGCAGAC	AGGTAAGCAAGGCAGATGGTGG	60
*Egr1*	TTCAACCCTCAGGCGGACAC	GTCTCCACCAGCACCTTCTCGT	60
*HPRT*	GCAGTCCCAGGGTGCGTG	GGCCTCCCATCTCCTTCAT	60
*Ywhaz*	AGGTTGCCGCTGGTGATGAC	GGCCAGACCCAGTCTGATAGGA	60
*Rpl13a*	CCTGGAGGAGAAGAGGAAAGAGA	TTGAGGACCTCTGTGTATTTGTCAA	60
*BTC*	GCGGAAAGGCCACTTCTCTAGG	TCATCACAGACACAGGAGGGCG	60
*EGF*	GCAGCTTCAGGACCACAACCA	AAACCATTCCCATCTGCTGGCT	60
*EPGN*	ACTCCAGAGGCTGACACAGGAC	GGAGTTTCGCTCTTGTCACCCA	60
*HB-EGF*	GTCTGTCTGCTGGTCATCGTGG	CCCAGCCGATTCCTTGAGCA	60
*EREG*	GGTTTCCATCTTCTACAGGCA	TGTCTTCTGTCTGAACTAAAGCTG	60
*AREG*	GATACTCGGCTCAGGCCATTATGC	TCAAATCCATCAGCACTGTGGTCC	60
*TGFα*	GCTGATACACTGCTGCCAGGTC	AGCAAGCGGTTCTTCCCTTCAG	60
*ADAM17*	TCGAGGGTGGATGAAGGAGAAGAG	GGACTGTTCCTGTCACTGCACTG	62
*ADAM10*	TCCACAGCCCATTCAGCAA	GCGTCTCATGTGTCCCATTTG	60
*ADAM8*	CACCAAAGCAGGTCATCAAGCCA	GCAACCTTTGGGCCAACAGCAC	62
*MMP-1*	CTGCCAAATGGGCTTGAAGCTGC	AGTTCTAGGGAAGCCAAAGGAGCT	62
*MMP-2*	CCTGACATTGACCTTGGCACCG	GTCCGCCAAATGAACCGGTCCT	62
*MMP-3*	ATTCAGTCCCTCTATGGACCTCCC	ACAGCATCAAAGGACAAAGCAGGA	58
*MMP-7*	TGGGAACAGGCTCAGGACTATCTC	TGACGCGGGAGTTTAACATTCCAG	62
*MMP-9*	TTCTTCTCTGGGCGCCAGGT	CCGCTGAACAGCAGCATCTTCC	58
